# Potential Association of the *CSMD1* Gene with Moderate Intellectual Disability, Anxiety Disorder, and Obsessive–Compulsive Personality Traits

**DOI:** 10.3390/ijms26094297

**Published:** 2025-05-01

**Authors:** Antonino Musumeci, Mirella Vinci, Simone Treccarichi, Donatella Greco, Biagio Rizzo, Angelo Gloria, Concetta Federico, Salvatore Saccone, Sebastiano Antonino Musumeci, Francesco Calì

**Affiliations:** 1 Oasi Research Institute-IRCCS, 94018 Troina, Italy; amusumeci@oasi.en.it (A.M.); mvinci@oasi.en.it (M.V.); streccarichi@oasi.en.it (S.T.); dgreco@oasi.en.it (D.G.); brizzo@oasi.en.it (B.R.); agloria@oasi.en.it (A.G.); musumeci@oasi.en.it (S.A.M.); cali@oasi.en.it (F.C.); 2 Department of Biological, Geological and Environmental Sciences, University of Catania, 95124 Catania, Italy; concetta.federico@unict.it

**Keywords:** complement activity, whole-exome sequencing, heterozygous variants, neurodevelopmental disorders

## Abstract

*CSMD1* is a gene involved in various biological processes and is highly expressed in the central nervous system, where it plays a key role in complement activity, brain circuit development, and cognitive function. It has been implicated as a susceptibility gene for schizophrenia and a causative factor in developmental epileptic encephalopathy, neurodevelopmental disorders, and intellectual disability. However, no MIM phenotype number has been assigned to *CSMD1* for a specific disorder. Here, we report an individual presenting with moderate intellectual disability, anxiety disorder, obsessive–compulsive personality traits, and facial dysmorphisms. Trio-based whole-exome sequencing (WES) identified two heterozygous *CSMD1* variants, c.8095A>G and c.5315T>C, both classified as variants of uncertain significance (VUS) according to ACMG criteria. Computational analysis using the DOMINO tool supported an autosomal recessive inheritance model for *CSMD1*. This study contributes to the growing evidence linking *CSMD1* to neurodevelopmental phenotypes, highlighting the need for further investigations to clarify its pathogenic role.

## 1. Introduction

Complement proteins play a crucial role in brain development and function, as widely documented. Complement signaling is involved in both neuroprotection and the pathogenesis of neurological diseases, with dysregulation contributing to various neurodevelopmental and neurodegenerative disorders [1,2]. Various proteins traditionally linked to the complement cascade in innate immunity have been found to play crucial roles in brain development. Notably, the classical complement cascade is crucial for normal postnatal synaptic pruning, with the C3b–CR3 interaction enabling microglial engulfment of low-activity synapses [3].

**Figure 1:**
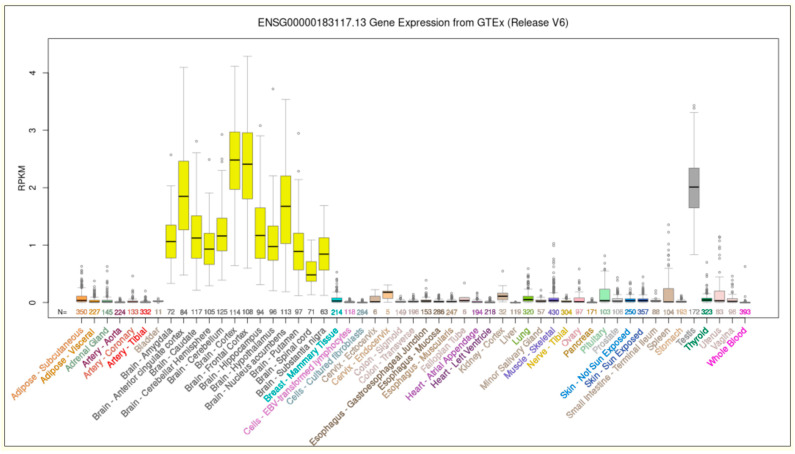
*CSMD1* gene expression in human healthy tissues. The expression level of the transcript ENSG00000183117.13 was obtained from 53 human tissues and 570 donors, using GTEx RNA-seq V6. RPKMs: Reads Per kilobase of transcript per million mapped reads. Data and image from the UCSC Genome Browser (http://genome.ucsc.edu, accessed on 9 March 2025).

CUB and Sushi domain-containing protein 1 (CSMD1) stands out as a key complement-regulatory protein with multiple domains, highly expressed in both the central nervous system (CNS) and epithelial tissues [4]. *CSMD1* is one of the largest genes in the human genome, spanning over 2 million base pairs and comprising 70 exons. It occupies almost the entire chromosomal band 8p24.3. The genomic sequence of *CSMD1* is highly GC-poor, with a GC content predominantly ranging between 35% and 40% (UCSC Genome Browser: https://genome.ucsc.edu, accessed on 9 March 2025). This nucleotide composition, which is typical of chromosomal regions harboring tissue-specific genes, is highly conserved during evolution [5,6]. Indeed, *CSMD1* is primarily expressed in the brain and testes, as confirmed by transcriptomic analyses (Figure 1).

Further investigations have shown that *CSMD1* is enriched in neurons, particularly in cortical and hippocampal regions, suggesting a potential role in synaptic function and neuronal connectivity [4,7]. Additionally, its expression in the testes correlates with stages of spermatogenesis [8], although its precise function in germ cell development remains to be elucidated.

The CSMD1 protein is characterized by a large membrane-bound structure that includes a short single transmembrane domain. This transmembrane region contains a potential tyrosine phosphorylation site, suggesting a role in regulating cellular functions. While the precise mechanisms of CSMD1 remain unclear, it has been implicated in various biological processes, including development, complement regulation, neurodevelopment, and cancer progression [9].

As documented, somatic mutations within this gene have been associated with various types of cancer [10,11,12]. Nevertheless, *CSMD1* was recently associated with cognitive function, complement activity and immediate episodic memory [13].

Several studies highlight the potential role of CSMD1, which is considered a statistically strong risk factor for schizophrenia [14,15,16,17]. In fact, low expression levels of *CSMD1* have been detected in peripheral blood mononuclear cells in individuals with schizophrenia, in comparison to healthy controls [17]. A previous study in mice showed that the disruption of *Csmd1* induced behaviors reminiscent of blunted emotional responses, anxiety, and depression. These observations suggest an influence of the *CSMD1* schizophrenia susceptibility gene on psychopathology and endophenotypes of the negative symptom spectra [18].

As recently documented, mice lacking Csmd1 exhibited increased levels of the complement component C3, greater colocalization of C3 with presynaptic terminals, fewer retinogeniculate synapses, and disrupted segregation of eye-specific retinal inputs to the visual thalamus during the critical period of complement-dependent circuit refinement [19]. In the central nervous system (CNS), C3 serves as a key effector with diverse functions, acting as a potent modulator of astrocyte activity and playing critical roles in inflammation, neurodegeneration, and neuroprotection [20,21,22,23].

This study aims to explore a potential association between *CSMD1* and the broad spectrum of symptoms observed in the examined individual harboring two genetic variants in compound heterozygosity, further supporting existing evidence in the literature regarding the gene’s pathogenicity.

## 2. Results

### 2.1. Clinical Data

The individual examined in this study is the child of non-consanguineous parents. The pregnancy progressed physiologically until the seventh month; at seven months and two weeks, during a routine check-up, a cesarean section was scheduled due to the absence of a fetal heartbeat. After birth, the patient was placed in an incubator and experienced sucking difficulties, failing to latch onto the breast. The birth weight was 2.6 kg, the length was 47 cm, and cyanosis at birth was reported. At nine days of life, pulmonary valve stenosis was diagnosed, followed by the detection of a small atrial septal defect. The patient began walking shortly after 15 months, started speech therapy at three years old, developed structured language around seven years, and achieved sphincter control at six years. Learning difficulties were reported. At ten months, episodes of loss of consciousness occurred, and valproic acid was administered until the age of four years. The mother reported frequent infectious and inflammatory episodes. Genetic analyses, including karyotyping, array-CGH, and MLL2 gene molecular analysis, were all negative. At clinical examination, the patient weighed 79 kg (75th–90th percentile), measured 166 cm in height (3rd–10th percentile), with a BMI of 28.73 kg/m^2^, indicating excess weight, and a head circumference of 57 cm (50th–97th percentile). Their general conditions were good, but notable dysmorphic features included a flat occiput, a square-shaped face, a prominent chin, downward-slanting palpebral fissures with mild eversion of the outer third of the lower eyelid, thick eyebrows, and slightly anteriorly rotated ears. Pulmonary, cardiac, abdominal, and genital examinations were unremarkable, and the patient exhibited an autonomous gait with normal muscle tone, trophism, and strength. On the Token Test, verbal comprehension was below the expected level for educational attainment, scoring more than two standard deviations below the mean.

### 2.2. Genetic Variant Detection

**Figure 2:**
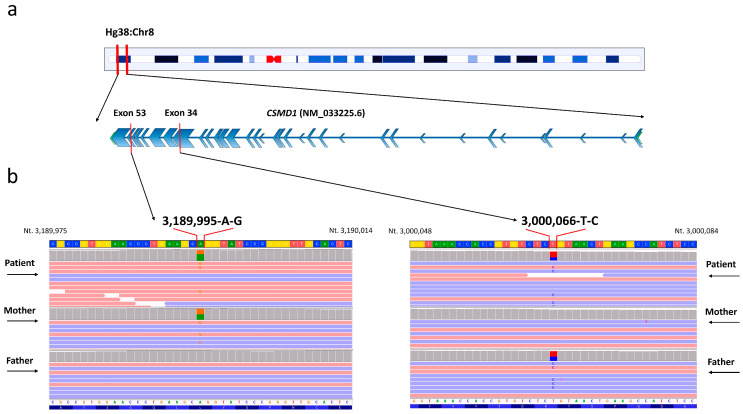
Next-generation sequencing for the identification of the c.5315T>C and c.8095A>G variants in compound heterozygosity within the *CSMD1* (NM_033225.6) gene: (**a**) graphical representation of the chromosomal localization of the *CSMD1* gene and the specific variants, located in exon 34 (chromosomal position 3,189,995 A>G, corresponding to c.5315T>C in the NM_033225.6 transcript) and exon 53 (chromosomal position 3,000,066 T>C, corresponding to c.8095A>G in the NM_033225.6 transcript); (**b**) graphical representation of the whole-exome sequencing (WES) analysis performed on the patient, his mother, and his father. The figure has been adapted from the Integrative Genomics Viewer (IGV) software version 2.19.4.

Whole-exome sequencing analysis identified the heterozygous variants c.5315T>C p.Leu1772Pro and c.8095A>G p.Arg2699Gly within the *CSMD1* (NM_033225.6) gene (Figure 2).

**Figure 3:**
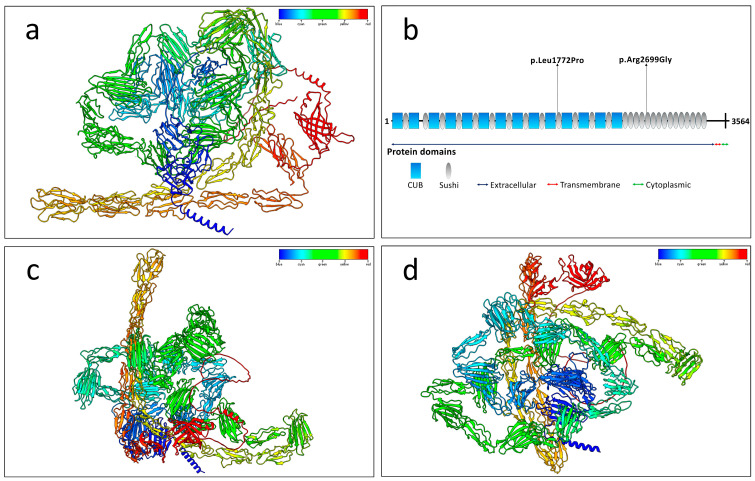
Structure prediction of the wild-type and both mutated CSMD1 proteins. The rainbow palette of color has been assigned from the starting amino acids (blue) to the final amino acids (red). (**a**) Structure prediction of the wild-type CSMD1 protein. (**b**) Graphical representation of the domain organization of the CSMD1 protein. This Figure has been adapted from a previous work [24]. (**c**) Structure prediction of the CSMD1 protein with the mutation p.Leu1772Pro. (**d**) Structure prediction of the CSMD1 protein with the mutation p.Arg2699Gly. All the structures of CSMD1 have been predicted using the AlphaFold algorithm and modeled using the software UCSF ChimeraX version 1.8.

The genetic variants were confirmed with Sanger sequencing (Supplementary Figure S1). The variant c.5315T>C was classified as “Uncertain significance”, according to the ACMG guidelines and following the criteria PP4, PM2, and PP3. With regard to the variant c.8095A>G, it was described as “Uncertain significance” following the criteria PP4, PM2, and PP3. The mutation p.Leu1772Pro was localized within the Sushi 10 domain (aa 1739-1800), while the mutation p.Arg2699Gly was localized within the Sushi 18 domain (aa 2678-2735) (Figure 3).

### 2.3. Protein Structure Prediction and Hydrogen Bonding Alterations

Protein model predictions for the wild-type and both mutated proteins revealed slight differences in the number of hydrogen bonds. Specifically, the wild-type protein formed a total of 2552 hydrogen bonds, while the p.Leu1772Pro mutant formed 2548 hydrogen bonds. In comparison, the p.Arg2699Gly mutant formed 2558 hydrogen bonds.

**Figure 4:**
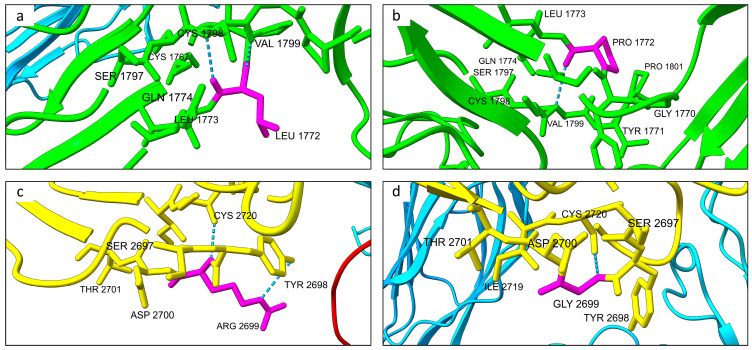
Close up of the hydrogen bonds’ interaction involving the wild-type Leu1772 and Arg2699 residues and the mutated Pro1772 and Gly2699. (**a**) Focus on the hydrogen bond interactions involving the wild-type residue Leu1772 of CSMD1 protein. As predicted, it engaged two hydrogen bonds with Val1779 at distances of 2.952 Å and 2.769 Å, respectively. (**b**) Close up on the mutated Pro1772 which engages a hydrogen bond interaction with the residue Val1799 at the distance of 3.234 Å. (**c**) Hydrogen bonds formed between the wild-type Arg2699 and both Tyr2698 (3.566 Å) Cys2720 (2.841 Å), respectively. (**d**) Interaction between the mutated Gly2699 and the residue Cys2720 (2.877 Å).

As predicted, the wild-type residue Leu1772 formed two hydrogen bonds with Val1779 at distances of 2.952 Å and 2.769 Å, respectively, while Arg2699 formed hydrogen bonds with Tyr2698 (3.566 Å) and Cys2720 (2.841 Å) (Figure 4). In contrast, the mutated Pro1772 formed only a single hydrogen bond with Val1779 (3.234 Å), and the mutated Gly2699 retained just one hydrogen bond with Cys2720 (2.877 Å), indicating a reduction in hydrogen bonding interactions upon mutation.

### 2.4. Structural Alignment and Stability Predictions

**Figure 5:**
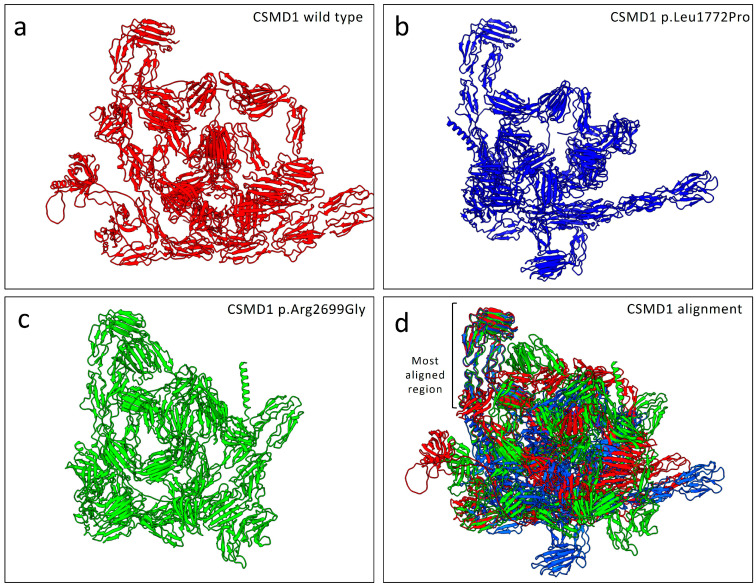
The structural alignment comparing the wild-type protein (red) with the mutated p.Leu1772Pro (blue) and p.Arg2699Gly (light green) CSMD1 proteins: (**a**) wild-type CSMD1 protein structure (red); (**b**) predicted structure of the p.Leu1772Pro mutant (blue); (**c**) predicted structure of the p.Arg2699Gly mutant (light green); and (**d**) superimposed structures showing alignment between wild-type and mutant proteins, with the square bracket indicating the most conserved structural region across all variants. Structural predictions were derived from AlphaFold models, and alignments were performed using UCSF ChimeraX (version 1.8).

The protein alignment comparing the wild-type structure model with both mutated structures showed notable differences between the wild-type and the mutated structures (Figure 5). Specifically, the alignment resulted in an RMSD of 1.157 Å for 560 pruned atoms. When considering all 3564 residues, the alignment yielded an overall RMSD of 44.211 Å.

Both MuPRO and mCSM tools predicted a decrease in protein stability as a consequence of the observed mutations. Specifically, MuPRO estimated a ΔΔG of −1.661 kcal mol^−1^ for the p.Leu1772Pro mutation and −1.914 kcal mol^−1^ for the p.Arg2699Gly mutation, indicating reduced stability. Similarly, mCSM predicted a ΔΔG of −0.892 kcal mol^−1^ and −0.347 kcal mol^−1^ for p.Leu1772Pro and p.Arg2699Gly, respectively, further supporting the destabilizing effect of these variants.

### 2.5. Predicted Structural Disorder

**Figure 6:**
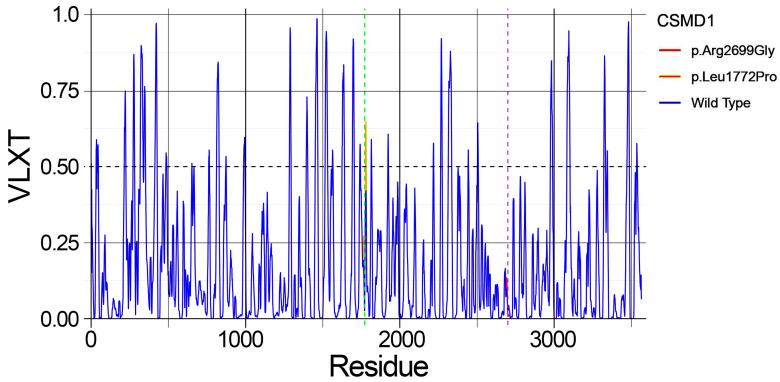
PONDR analysis predicted the structural disorder of the CSMD1 protein as a consequence of both identified variants. The blue line represents the VLXT score variation in the wild-type protein, while the orange line indicates the VLXT score variation in the p.Leu1772Pro mutant, showing an increased structural disorder (VLXT score > 0.50) affecting residues 1777–1782. The green dashed line marks the mutation site at position 1772. The red line represents the VLXT score variation in the p.Arg2699Gly mutant, which did not exhibit any notable differences compared to the wild-type protein. The purple dashed line indicates the mutation site at position 2699.

PONDR analysis predicted a significant increase in structural disorder as a consequence of the p.Leu1772Pro mutation in the CSMD1 protein compared to the wild-type (Figure 6).

Specifically, the mutation resulted in an elevated disorder, indicated by a VLXT score greater than 0.50, affecting residues 1777–1782, which was not observed in the wild-type protein. In contrast, the PONDR disorder prediction for the mutation p.Arg2699Gly showed no notable differences compared to the wild-type protein.

### 2.6. Allele Frequency and Evolutionary Conservation

According to the GnomAD database, both identified genetic variants exhibited low allele frequencies. Specifically, the c.5315T>C variant had a frequency of 0.0000006252, detected in only 1 allele out of 1,599,564 total alleles, in a female of European non-Finnish ancestry. Similarly, the c.8095A>G variant was found in 4 alleles (1 in an individual of African ancestry and 3 in European non-Finnish females) out of 1,603,262 total alleles, corresponding to an allele frequency of 0.000002738. Additionally, the DOMINO tool indicated a highly probable autosomal recessive inheritance pattern for the *CSMD1* gene, with a score of 0.254.

**Figure 7:**
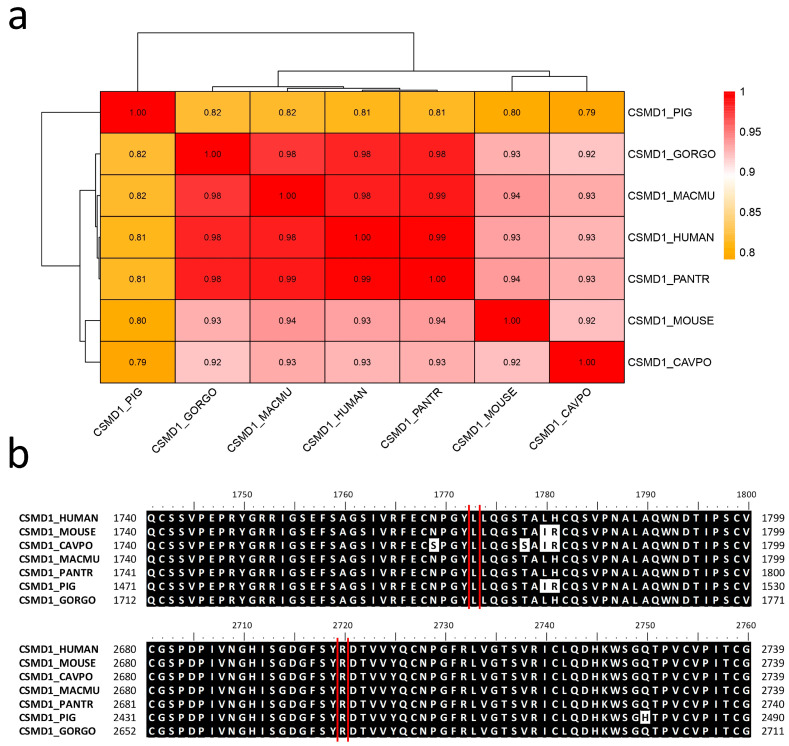
Sequence identity analysis of the CSMD1 protein across different species, including *Homo sapiens* (HUMAN), *Sus scrofa* (PIG), *Gorilla gorilla* (GORGO), *Macaca mulatta* (MACMU), *Pan troglodytes* (PANTR), *Mus musculus* (MOUSE), and *Cavia porcellus* (CAVPO). (**a**) Heatmap depicting the sequence identity of the CSMD1 protein in *Homo sapiens* compared to the six aforementioned species. (**b**) Graphical representation of the amino acid positions affected by the mutations, highlighting the wild-type Leu1772 and Arg2699 residues. These specific residues are marked with red lines.

The sequence alignment of the human CSMD1 protein with those of other mammals revealed a sequence identity ranging from 81% with *Sus scrofa* to 99% with *Pan troglodytes* (Figure 7). Both the wild-type Leu1772 and Arg2699 residues are highly conserved across mammalian species, indicating their potential functional significance.

## 3. Discussion

In this study, we report an individual displaying moderate intellectual disability, anxiety disorder, obsessive–compulsive personality traits, and facial dysmorphisms. WES analysis performed in the examined patient and both healthy parents identified the compound heterozygous genetic variants c.5315T>C and c.8095A>G. Both the variants were described as “uncertain significance” following the ACMG criteria. According to the DOMINO database, the *CSMD1* gene follows an autosomal recessive inheritance pattern. Data from the GnomAD database indicate that both variants have a very low allele frequency in the human population, suggesting that these mutation sites are not under strong selective pressure. To date, this gene does not have an MIM phenotype entry in the OMIM database associated with a specific condition. As recently documented, biallelic variants found in this gene are implicated in a neurodevelopmental disorder with intellectual disability and variable cortical malformations [24]. In the individual examined in this study, notable dysmorphic facial features included a flat occiput, a square-shaped face, a prominent chin, downward-slanting palpebral fissures with mild eversion of the outer third of the lower eyelid, thick eyebrows, and slightly anteriorly rotated ears. As reported, the intronic variant rs2740931 has been associated with the learning phase of verbal memory tests [13], and our patient demonstrated verbal comprehension below the expected level for their educational attainment. Furthermore, he underwent speech therapy at three years old, and learning difficulties were reported.

This study aims to explore a potential association between CSMD1, and the wide array of symptoms observed in the examined patient. However, we cannot rule out the possibility that other undetected genetic variants or epigenetic modifications may contribute to the patient’s phenotype. In this context, we emphasize that both karyotype analysis and array CGH did not reveal chromosomal abnormalities in the examined individual.

As indicated in the gene expression database GTEx, CSMD1 shows high expression patterns in the human brain, specifically, in the amygdala, anterior cingulate cortex, frontal cortex, hippocampus, hypothalamus, nucleus accumbens (basal ganglia), and spinal cord.

**Figure 8:**
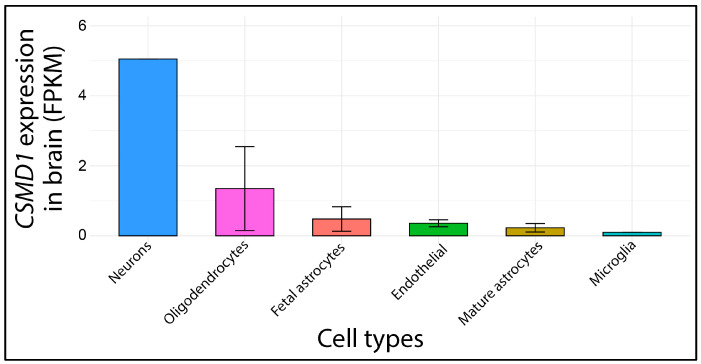
*CSMD1* expression profile in human brain across different cell types. Data are expressed in fragments per kilo base of transcript per million mapped fragments (FPKMs). The dataset used in this study was obtained from previously published work [25] and is publicly available in the BrainRNAseq repository (https://brainrnaseq.org) (accessed on 7 February 2025). Data analysis was performed using R Studio (version 3.4.3) with the ggplot2 package.

On the other hand, as documented in the BrainRNAseq database, CSMD1 displays the highest expression pattern in neurons (Figure 8).

**Figure 9:**
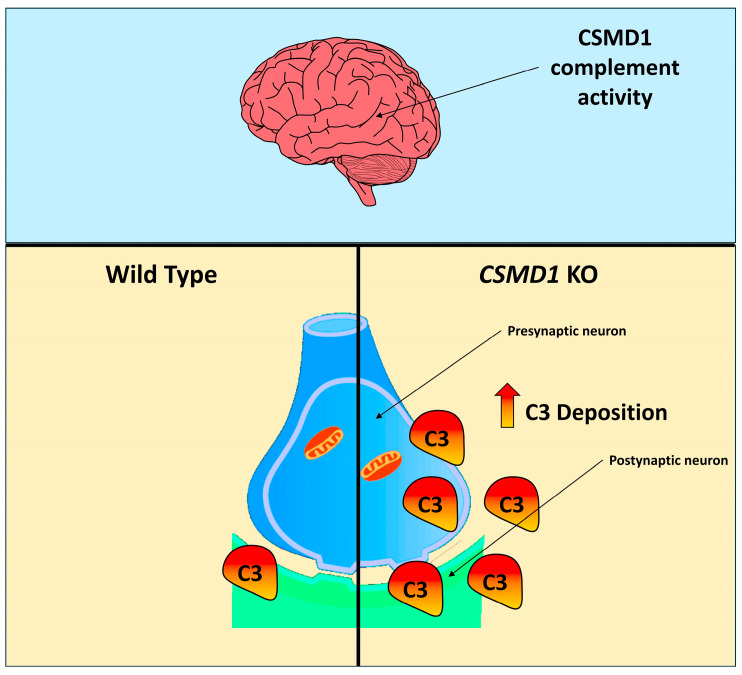
Synaptic model of complement activity regulated by *CSMD1*. As previously documented, knockout of the *CSMD1* gene in mice led to the dysregulation of complement activity, resulting in increased and uncontrolled C3 deposition [19]. This figure has been adapted from a previous study [19].

Within this context, we consider it plausible that the mutation altered the gene expression profile of *CSMD1* across the human brain, potentially disrupting its complement inhibition activity. Furthermore, supported by recent documentation [26], the dysregulation of *CSMD1* complement components may contribute to psychosis and is associated with cognitive function. Also, as recently documented, *CSMD1* has been described as a causative gene of developmental and epileptic encephalopathy and generalized epilepsies [27]. It is worth mentioning that, despite *CSMD1* having been described as a candidate susceptibility gene for schizophrenia, its specific role in neurodevelopmental disorders is unclear [15,16]. To elucidate this mechanism, a previous model was proposed and assessed through experimental studies in mice [19]. This model involved the inhibition of the complement cascade in neural tissues mediated by *CSMD1*. As demonstrated, knockout of the *CSMD1* gene led to increased complement activity in the brain, resulting in the accumulation of the complement protein C3, which, in turn, contributed to the development of psychiatric disorders. Figure 9 summarizes the previously mentioned model.

According to the HGMD database, 2/3 of the 160 genetic variants identified in the *CSMD1* gene have been associated with neurodevelopmental disorders. Furthermore, most of the annotated variants have been classified as likely disease-causing mutations (DM?) for their uncertain significance.

The mutations p.Leu1772Pro and p.Arg2699Gly were localized within the Sushi 10 (aa 1739-1800) and 18 domains (aa 2678-2735), respectively (Figure 3). As annotated in ProSite database, these specific domains (PRU00302) are involved in many recognition processes, including the binding of several complement factors to fragments C3b and C4b. Furthermore, both mutations occurred within the extracellular protein region (aa 27-3487). It is worth noting that both mutated amino acids are highly conserved across different species (Figure 7b), indicating their evolutionary significance. This strong conservation suggests that these residues play a crucial functional role, and any alteration may disrupt essential protein interactions or biological processes. Evolutionary conservation often implies selective pressure to maintain the integrity of these amino acids, further supporting their potential impact on protein function when mutated.

Protein structure predictions using the AlphaFold algorithm revealed differences in both mutated structures compared to the wild-type protein. Protein model predictions also indicated slight differences in the number of hydrogen bonds. Specifically, the total number of hydrogen bonds showed minor variations compared to the wild-type structure. However, notable differences were observed at the mutated residues Pro1772 and Gly2699 when compared to the wild-type residues Leu1772 and Arg2699. In particular, both mutations resulted in the loss of one hydrogen bond at the mutated residue compared to the respective wild-type amino acid (Figure 3). Despite the low variation in the total number of hydrogen bonds, structural alignment of both mutated proteins with the wild-type protein revealed notable differences in folding. Across the total of 3564 residues, only 560 amino acids were aligned across all three structures.

As predicted by MuPRO and mCSM tools, both the observed mutations caused a destabilizing effect within the protein structure. On the other hand, the prediction of the structural disorder carried out with PONDR, predicted a notable increase in the protein disorder comprising residues 1777–1782, as results of the mutation p.Leu1772Pro.

This work strengthens the foundations related to the potential association, corroborated by the literature, between CSMD1 and neuropsychiatric disorders. Nevertheless, we emphasize that functional studies are imperative for this purpose and for assigning an MIM phenotype code associating this gene to a specific phenotype.

## 4. Materials and Methods

### 4.1. Library Preparation and Whole-Exome Sequencing (WES)

Genomic DNA was obtained from the patient and their parents’ peripheral blood leukocytes, as previously described [28]. The preparation of libraries (TRIOS) and exome enrichment were carried out using the Agilent SureSelect V7 Kit (Santa Clara, CA, USA), in accordance with the manufacturer’s instructions. The sequencing run was performed by the Illumina HiSeq 3000 instrument (San Diego, CA, USA). The method adopted enabled the achievement of 97% of regions covered at least 20×. The unveiled variants were filtered based on (i) a recessive/de novo/X-linked pattern of inheritance and (ii) allele frequencies (minor allele frequency, MAF) <1%, using, as a reference, the following genomic datasets: 1000 Genomes, ESP6500, ExAC, GnomAD. The reference genome used for this study was HG38. Both variants were confirmed through conventional Sanger sequencing using the BigDyeTM Terminator v1.1 Cycle Sequencing Kit (Life Technologies, Carlsbad, CA, USA) with the SeqStudio Genetic Analyzer instrument (Thermo Fisher Scientific, Waltham, MA, USA).

### 4.2. Variant Identification and Filtering

The variants were searched on the Human Gene Mutation Database (HGMD Professional 2024.4) (https://www.hgmd.cf.ac.uk/ac/index.php) (accessed on 7 February 2025). The notation “(DM?)” indicates a variant classified as likely disease-causing but with uncertain pathogenicity, as documented in [29]. Furthermore, they were filtered using Franklin (Genoox, Tel-Aviv, Israel) (https://franklin.genoox.com) (accessed on 4 February 2025). The described variants were classified according to the “American College of Medical Genetics” (ACMG) guidelines [30].

### 4.3. Inheritance Pattern and Allele Frequencies

The inheritance pattern of the *CSMD1* gene was determined using the DOMINO web server (https://domino.iob.ch/) (accessed on 7 February 2025). Allele frequencies of both variants were retrieved from the GnomAD database v4.1.0 (https://gnomad.broadinstitute.org/) (accessed on 7 February 2025).

### 4.4. Gene Expression and Functional Domain Analysis

The genetic expression of the *CSMD1* gene in human tissues was analyzed using GTEx (https://www.gtexportal.org/) (accessed on 7 February 2025). The functional domains of the CSMD1 protein were examined using the Uniprot (https://www.uniprot.org/) (accessed on 7 February 2025) and Prosite (https://prosite.expasy.org/) (accessed on 7 February 2025) databases.

### 4.5. Impact of Mutations on Protein Structure

To assess the thermodynamic impact of the identified mutations on the CSMD1 protein structure, the MuPRO tool (https://mupro.proteomics.ics.uci.edu/) (accessed on 7 February 2025) and mCSM (https://biosig.lab.uq.edu.au/mcsm/) (accessed on 7 February 2025) were used. Additionally, the PONDR tool (http://pondr.com/) (accessed on 7 February 2025) was utilized to study the stability changes resulting from the mutations within the CSMD1 structure.

### 4.6. Protein Structure Prediction and Modeling

The structures of the CSMD1 proteins were predicted using the AlphaFold server (https://alphafoldserver.com/) (accessed on 7 February 2025), selecting the “best model”. These structures were modeled using UCSF ChimeraX software version 1.8. The predicted alignment error (PAE) heatmaps for the three top-ranked structural models are presented in Supplementary Figure S2.

### 4.7. Sequence Alignment and Phylogenetic Analysis

The sequence alignment of the human CSMD1 protein with six mammalian species (*Sus scrofa*, *Macaca mulatta*, *Mus musculus*, *Pan troglodytes*, *Gorilla gorilla*, and *Cavia porcellus*) was performed using BioEdit version 7.2 (Software Informer, https://bioedit.software.informer.com/7.2/ (accessed on 7 February 2025)) [31]. This software was also used to calculate the identity matrix. The protein alignment data were processed to generate a heatmap with a dendrogram using R Studio version 3.4.3 and the pheatmap package.

### 4.8. Brain Expression Profile Analysis

The expression profile of *CSMD1* across various human brain organs was retrieved from the Brain RNAseq database (https://brainrnaseq.org/) (accessed on 7 February 2025). The dataset included the following cell populations: fetal astrocytes (n = 6), astrocytes mature (n = 12), endothelial (n = 2), microglia (n = 3), neurons, and oligodendrocytes (n = 5). Data visualization was performed using the ggplot2 package in R Studio version 3.4.3, generating a comparative expression barplot.

## 5. Conclusions

Whole-exome sequencing (WES) analysis identified two genetic variants, c.5315T>C and c.8095A>G, in the *CSMD1* gene in compound heterozygosity in an individual presenting with moderate intellectual disability, anxiety disorder, obsessive–compulsive personality traits, and facial dysmorphisms. While intronic variants of *CSMD1* have previously been associated with schizophrenia, biallelic exonic variants have been linked to neurodevelopmental disorders. This study further supports the potential association between *CSMD1* and neurodevelopmental phenotypes. We hope that this work will inspire further research aimed at elucidating the molecular mechanisms of *CSMD1*, which remain largely unknown. Additionally, we anticipate that an MIM phenotype number will eventually be assigned to *CSMD1*, associating it with neurodevelopmental disorders and an autosomal recessive inheritance pattern.

## Data Availability

The data related to this study are presented in the main text.

## Supplementary Materials

The following supporting information can be downloaded at https://www.mdpi.com/article/10.3390/ijms26094297/s1.

## Abbreviations

The following abbreviations are used in this manuscript:

ACMG	American College of Medical Genetics
CSMD1	CUB and Sushi domain-containing protein 1
HGMD	Human Gene Mutation Database
WES	Whole-exome sequencing
