# Supplementary material for: Potential Association of the CSMD1 Gene with Moderate Intellectual Disability, Anxiety Disorder, and Obsessive–Compulsive Personality Traits
Source: Int J Mol Sci. 2025 May 1;26(9):4297. doi: 10.3390/ijms26094297 (PMC12072550; doi:10.3390/ijms26094297)
Supplement: Supplementary file 1 [file ijms-26-04297-s001.zip › ijms-3567221-supplementary.pdf]

Supplementary figures

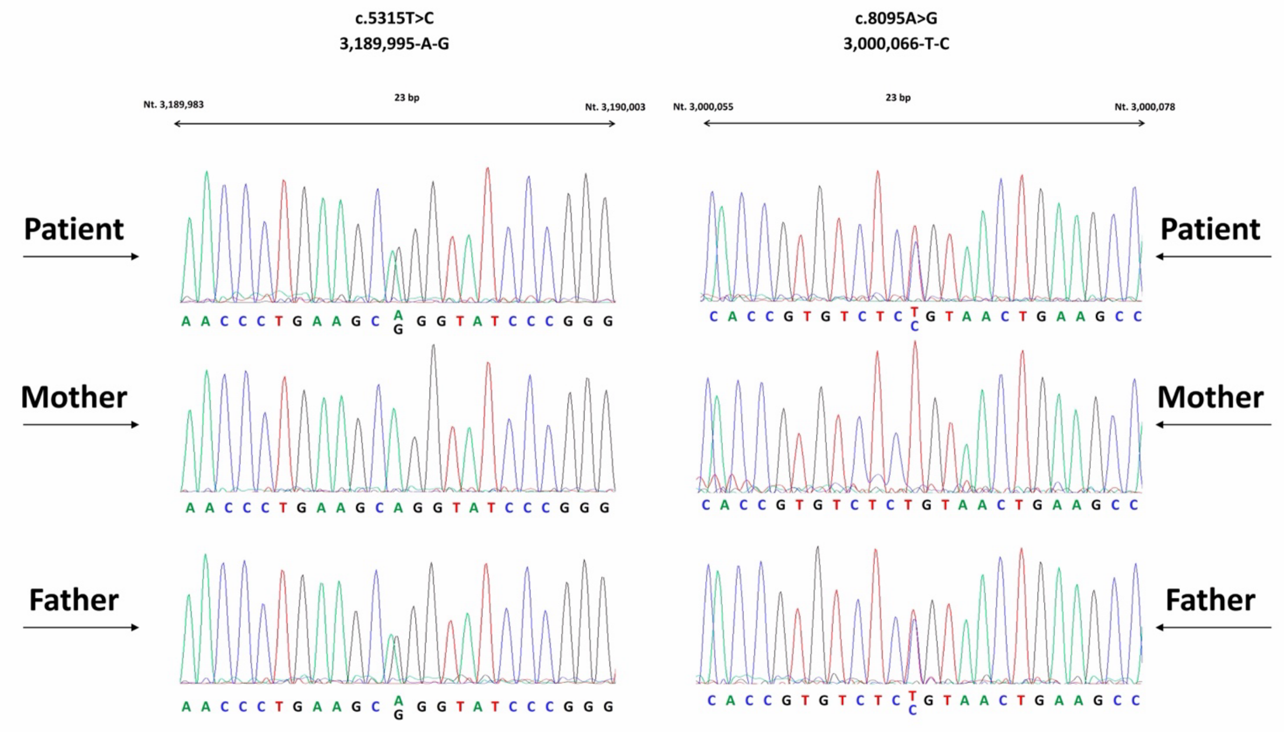

Figure S1. Sanger sequences for the c.5315T>C and c.8095A>G variants within the *CSMD1* gene.

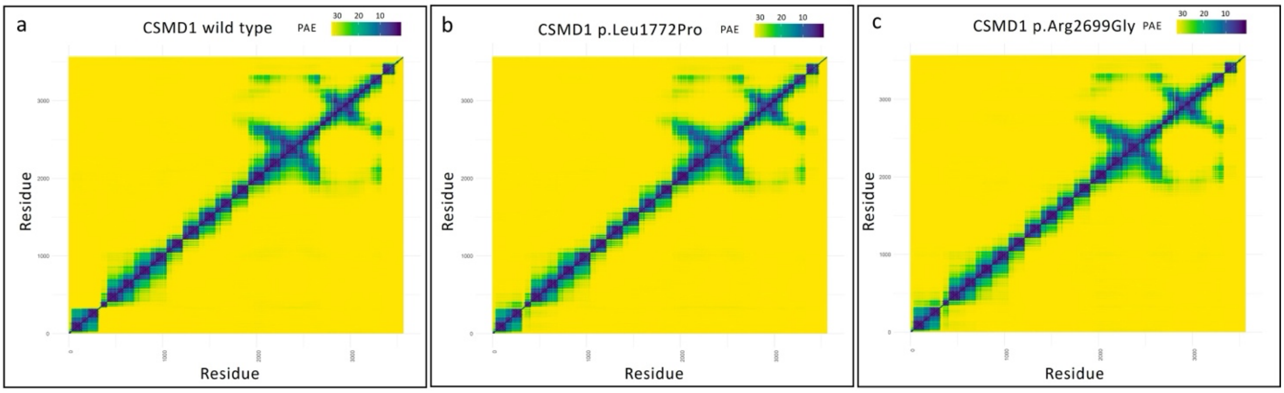

Figure S2. Predicted alignment error (PAE) heatmaps for the three top-ranked structural models: (a) selected wild-type CSMD1 model, (b) mutant variant p.Leu1772Pro and (c) mutant variant p.Arg2699Gly).
